# Successful Endoscopic Submucosal Dissection for Giant Inflammatory Fibroid Polyp in Terminal Ileum

**DOI:** 10.1002/deo2.70177

**Published:** 2025-07-24

**Authors:** Sayuri Watanabe, Yuki Nakajima, Masato Aizawa, Jun Wada, Kakeru Otomo, Goro Shibukawa, Tadayuki Takagi, Kenichi Utano, Osamu Suzuki, Kazutomo Togashi

**Affiliations:** ^1^ Department of Gastroenterology Aizu Medical Center Fukushima Medical University Fukushima Japan; ^2^ Department of Radiology Aizu Medical Center Fukushima Medical University Fukushima Japan; ^3^ Department of Diagnostic Pathology Aizu Medical Center Fukushima Medical University Fukushima Japan

**Keywords:** colonoscopy | endoscopic submucosal dissection | giant polyp | inflammatory fibroid polyp | terminal ileum

## Abstract

A 55‐year‐old woman presented with postprandial abdominal pain and diarrhea. Contrast‐enhanced abdominal computed tomography revealed a large tumor in the ileocecal region. Colonoscopy demonstrated a pedunculated polyp originating from the terminal ileum, intermittently prolapsing into the cecum with a stalk‐like base. Biopsy specimens showed nonspecific inflammatory changes. Initial hot snare polypectomy was unsuccessful due to the polyp's large size and mobility. Therefore, endoscopic submucosal dissection using the underwater pocket‐creation method was performed, with the polyp stabilized using a traction device anchored to its apex and the opposite side of the ileocecal valve. This technique enabled safe resection of the lesion from its broad stalk. Although marked submucosal fibrosis was observed beneath the lesion, en bloc resection was successfully completed without perforation in 63 min. Retrieval of the resected specimen via conventional endoscopic methods was unsuccessful due to difficulty passing through the hepatic flexure. Instead, the specimen was retrieved following natural elimination the next day. The resected specimen was a prolate spheroid measuring 62 × 40 × 22 mm. Histopathological examination confirmed an inflammatory fibroid polyp (IFP), consisting of edematous stroma with dense inflammatory cell infiltration. The patient resumed oral intake on postoperative day 2 and had an uneventful recovery. Follow‐up colonoscopy at 6 months revealed no residual or recurrent lesion. To our knowledge, this case represents the largest IFP of the small intestine ever resected endoscopically. For a giant, mobile lesion in the terminal ileum, the combination of the pocket‐creation method, underwater technique, and lesion anchoring was an effective strategy.

## 1 Introduction

Inflammatory fibroid polyps (IFPs) can develop throughout the gastrointestinal tract, with the small intestine being the second most common site after the stomach [1]. They are usually found in middle‐aged to elderly patients and may present with nonspecific symptoms or as causes of gastrointestinal bleeding or obstruction. Histologically, they are characterized by spindle cell proliferation, eosinophilic infiltration, and a characteristic perivascular onion‐skin pattern. IFP of the small intestine is often detected due to intussusception or bleeding [2, 3, 4, 5, 6, 7]. We report a case of a giant IFP in the terminal ileum causing obstructive symptoms, which was successfully treated with endoscopic submucosal dissection (ESD).

## 2 Case Presentation

**FIGURE 1:**
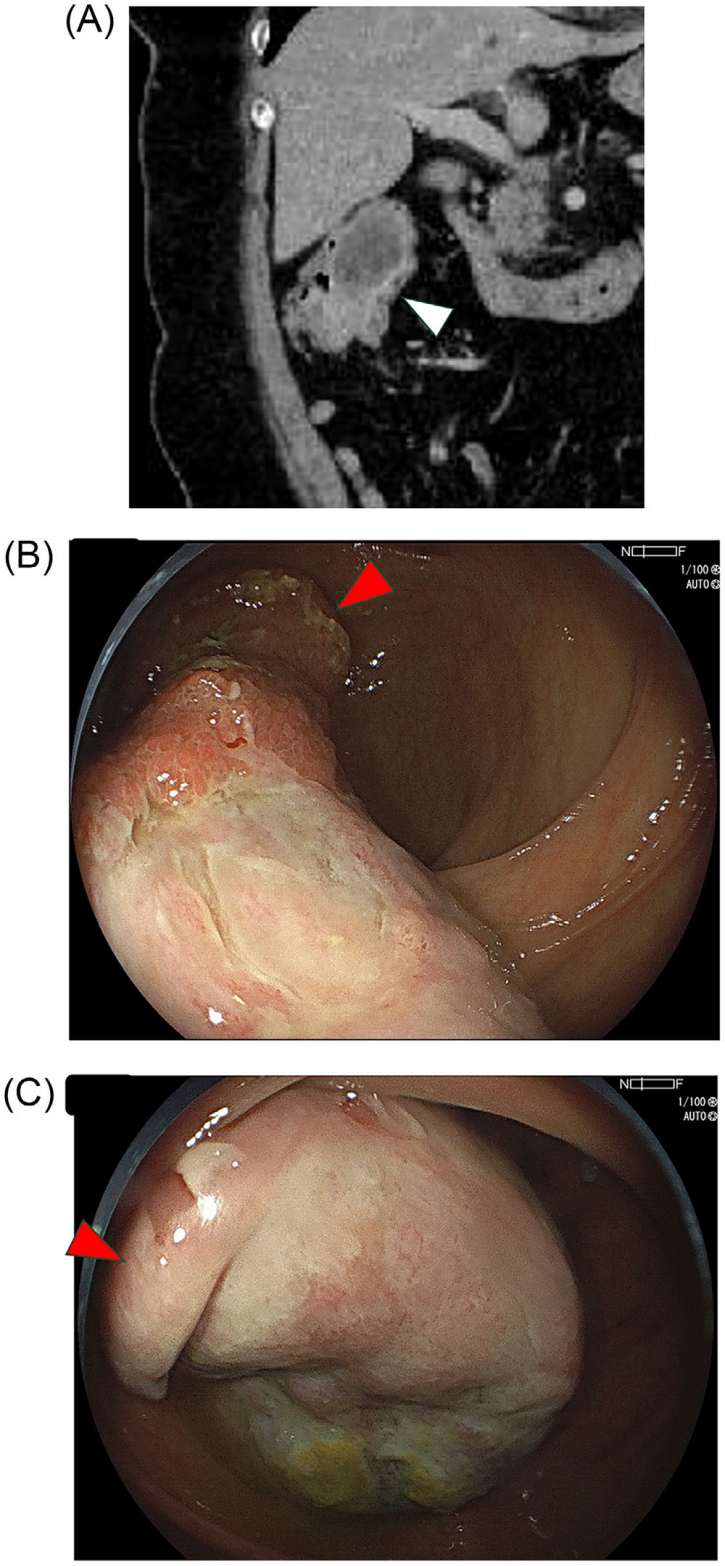
(A) Coronal computed tomography (CT) image. A low‐density lesion with an irregularly enhancing capsule (arrowhead) is visible in the right abdomen, connected to the ascending colon and terminal ileum. (B) Red arrowheads indicate the ileocecal valve. A giant ileal lesion could not be visualized in its entirety at a single glance. (C) Red arrowheads indicate the ileocecal valve. The lesion occasionally migrated into the terminal ileum. Its head was covered with a rough surface, inconsistent with a subepithelial lesion.

A 55‐year‐old woman presented with postprandial abdominal pain and diarrhea. She had been receiving methotrexate for rheumatoid arthritis for the past 5 years. Contrast‐enhanced abdominal computed tomography (CT) revealed an ileocecal mass measuring up to 65 mm in diameter, associated with intussusception (Figure 1A). Colonoscopy demonstrated a large pedunculated polyp originating from the terminal ileum, intermittently prolapsing into the cecum. The lesion had a stalk‐like base but was not immediately recognizable at first glance (Figure 1B,C and Video S1). Histological examination of biopsy specimens revealed inflammatory changes.

**FIGURE 2:**
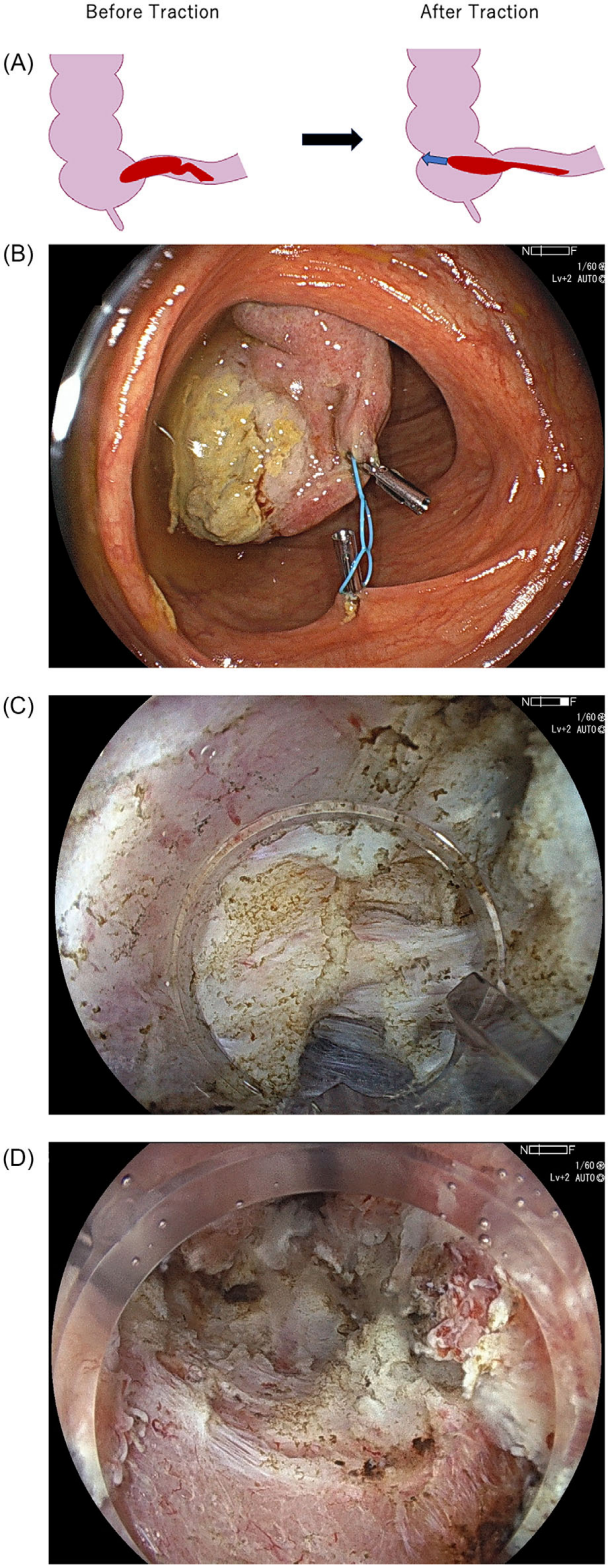
(A) Schematic illustration showing the polyp before and after traction of its head using a traction device (indicated by the blue arrow). The giant polyp, with a long and broad stalk, was initially highly mobile within the ileocolic area. The head of the polyp was stabilized by anchoring the traction device to its apex and to the opposite side of the ileocecal valve. (B) The polyp head was pulled into the ascending colon using a traction device, which was attached to the apex of the polyp and anchored to the opposite side of the ileocecal valve. (C) Submucosal dissection was performed under saline immersion. Marked fibrosis was observed directly beneath the lesion. (D) On close inspection, ulceration involved approximately 30% of the circumference after endoscopic submucosal dissection (ESD).

Endoscopic treatment was planned using a standard video endoscope (EC‐760ZP‐V/M endoscope; Fujifilm). An initial attempt at hot snare polypectomy was unsuccessful due to the polyp's large size and mobility. Specifically, when the endoscope was advanced close to the lesion base, the stalk‐like pedicle shifted position, making it difficult to maintain a stable field of view. Consequently, we opted to perform ESD after stabilizing the polyp, as shown in Figure 2A.

Using a traction device (multi‐loop traction device, Boston Scientific) attached to the top of the polyp, we pulled the polyp head into the ascending colon and maintained this position by anchoring the device to the opposite side of the ileocecal valve (Figures 2A and 2B). Sodium alginate mixed with indigo carmine and epinephrine was injected into the submucosa beneath the lesion base, and ESD was initiated using a knife (Flush knife BT‐S, 1.5 mm; Fujifilm) with the pocket‐creation method (PCM) under saline immersion. Severe submucosal fibrosis and abundant vasculature were observed beneath the lesion. Despite the limited working space within the terminal ileum, the combined use of PCM, the underwater technique, and anchoring of the lesion allowed for a stable visual field and sufficient maneuverability of the endoscope (Figure 2C). En bloc dissection was successfully completed without intraoperative perforation in 63 min (Video S1). The mucosal defect following ESD extended to approximately 30% of the ileal luminal circumference (Figure 2D). Accordingly, we considered the risk of post‐procedural stricture to be low and did not implement any preventive measures, including local triamcinolone injection or oral steroid administration.

**FIGURE 3:**
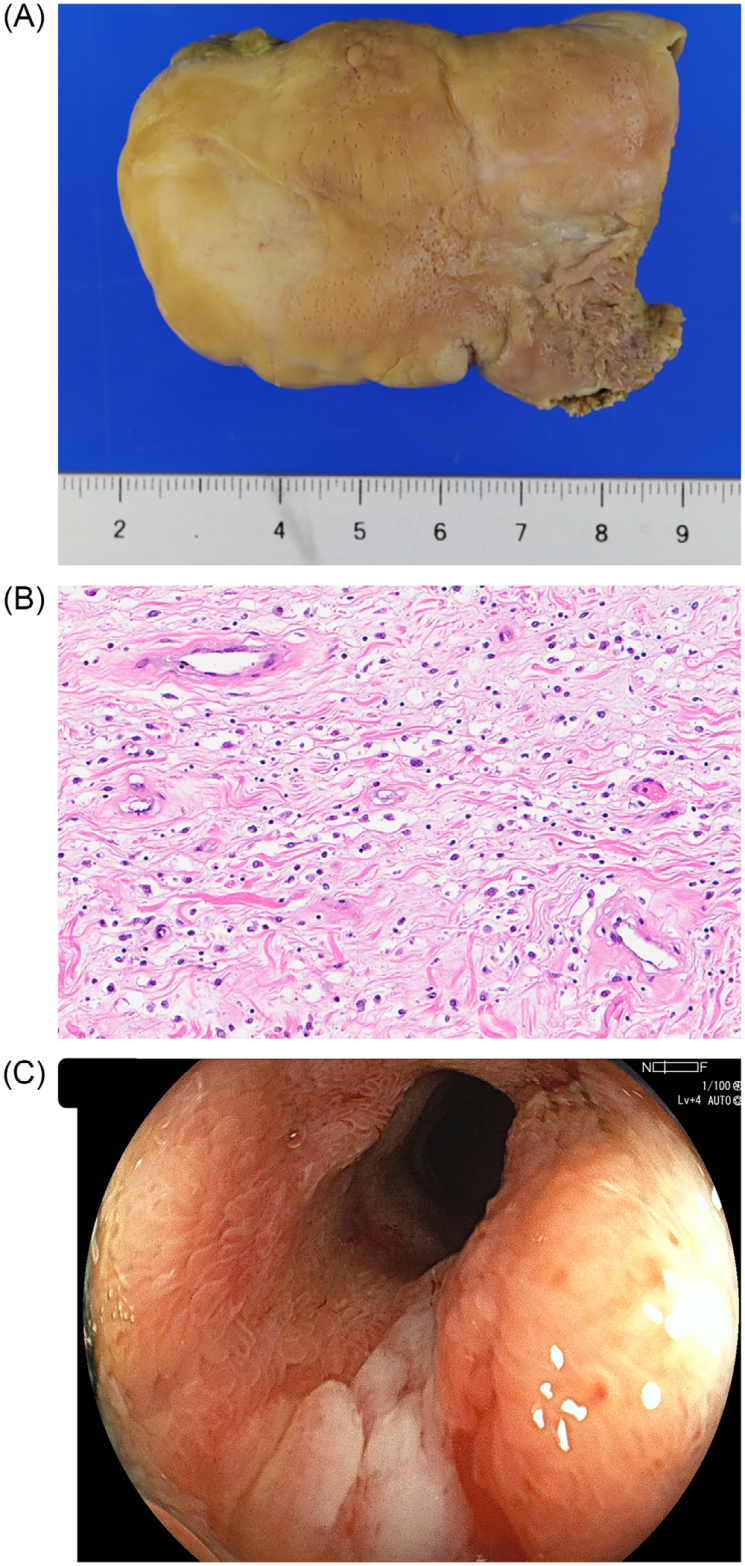
(A) The resected specimen was a prolate spheroid measuring up to 62 mm in its longest dimension. (B) Histopathological examination revealed an inflammatory polyp with edematous stroma and prominent infiltration of inflammatory cells, including neutrophils, eosinophils, and lymphocytes. (C) Endoscopic view 6 months after resection. No residual lesion was observed, but a linear ulceration remained at the resection site.

Retrieval of the resected specimen using conventional methods, such as a retrieval net, was unsuccessful, primarily due to difficulty in passing through the hepatic flexure, rather than the specimen's inability to fit into a retrieval device. Instead, we opted for natural elimination [8], and the specimen was collected on the first postoperative day. The resected specimen was a prolate spheroid measuring 62 × 40 × 22 mm with a thick, short stalk approximately 2 cm in maximum diameter. It was retrieved with minimal crushing or tissue damage (Figure 3A). Histopathological examination showed an IFP composed of edematous stroma with prominent infiltration of inflammatory cells, including neutrophils, eosinophils, and lymphocytes (Figure 3B). However, the characteristic perivascular onion‐skin pattern typically seen in IFPs was not observed in any area.

The patient resumed oral intake on the second postoperative day, had an uneventful recovery, and was discharged on the fourth postoperative day. Following resection, the symptoms of intestinal obstruction completely resolved. At the six‐month follow‐up, colonoscopy revealed no residual lesion but showed linear ulceration at the resection site (Figure 3C), indicating the need for periodic surveillance.

## 3 Discussion

According to previous reports [2, 3, 4, 5, 6, 7, 9], the majority of IFP in the small intestine have been surgically resected. Since the first report by Wolff et al. [2]. In 2008, a total of 19 small intestinal IFPs, including the present case, were resected endoscopically, as shown in Table 1. The median patient age was 62 years, ranging from 39 to 78 years. There was no apparent gender predominance (11 females and eight males). Except for one duodenal lesion, all 18 remaining lesions were located in the ileum. The median lesion size was 15 mm, with a range of 5–62 mm. The largest small intestinal IFP previously resected endoscopically measured 40 mm and was located in the duodenal bulb [9]. Although an ectopic pancreatic lesion measuring 75 mm in the terminal ileum was resected via endoscopic mucosal resection [10], the present case represents the largest confirmed IFP of the small intestine successfully removed using an endoscopic approach.

**TABLE 1:** Previously reported cases of small intestinal inflammatory fibroid polyps resected endoscopically, including the present case.

Case no.	Author	Publication Year	Age	Sex	Location	Size	Morphology	Resection method
1	Wolff et al. [2]	2009	73	Female	Distal ileum	20	Pedunculated	Snare polypectomy
2	Yoon et al. [3]	2012	39	Male	Distal ileum	25	Pedunculated	EMR
3	Laskaratos et al. [4]	2016	70	Female	Distal ileum	30	Pedunculated	EMR
4	Takeshita et al. [5]	2016	64	Male	Distal ileum	20	Sessile	EMR
5	Inayat et al. [9]	2018	52	Male	Duodenum	60	Pedunculated	ESD
6	Tang et al. [6]	2023	50	Female	Terminal ileum	8	Sessile	ESD
7	Tang et al. [6]	2023	54	Female	Terminal ileum	15	Sessile	ESD
8	Tang et al. [6]	2023	49	Male	Terminal ileum	8	Sessile	ESD
9	Tang et al. [6]	2023	68	Male	Terminal ileum	10	Sessile	ESD
10	Tang et al. [6]	2023	42	Female	Terminal ileum	12	Sessile	ESD
11	Tang et al. [6]	2023	51	Female	Terminal ileum	8	Sessile	ESD
12	Tang et al. [6]	2023	68	Female	Terminal ileum	10	Sessile	ESD
13	Tang et al. [6]	2023	70	Female	Terminal ileum	18	Sessile	ESD
14	Tang et al. [6]	2023	62	Male	Terminal ileum	5	Sessile	ESD
15	Tang et al. [6]	2023	78	Female	Terminal ileum	12	Sessile	ESD
16	Tang et al. [6]	2023	54	Male	Terminal ileum	5	Sessile	ESD
17	Tang et al. [6]	2023	67	Female	Terminal ileum	15	Sessile	ESD
18	Matsubara et al. [7]	2024	78	Male	Distal ileum	15	Sessile	EMR
19	Present case	2025	55	Female	Terminal ileum	62	Pedunculated	ESD

Abbreviations: EMR, endoscopic mucosal resection; ESD, endoscopic submucosal dissection.

This successful ESD can be attributed to precise preoperative diagnosis, the selection of appropriate ESD strategies, and substantial endoscopic expertise. Given the histologically confirmed benign nature of the lesion, endoscopic resection was considered a viable option despite its size. Since the terminal ileum is a narrow segment with limited endoscope maneuverability, the combination of PCM, the underwater technique, and lesion anchoring proved to be effective strategies for overcoming these limitations. By creating a submucosal pocket under saline immersion and applying traction to the lesion, we were able to maintain a stable and adequate working space. Notably, the underwater technique minimized the risk of overinsufflation by using water pressure instead of air, thereby enhancing visibility and improving scope control in the confined ileal lumen. In addition, anchoring the lesion not only reduced the mobility of the stalk‐like base but also prevented torsion, facilitating a consistent and direct approach to the dissection plane. Finally, the team's extensive experience with colorectal and gastric ESD played a crucial role in the successful execution of this procedure.

However, retrieval of the large specimen posed a significant challenge, emphasizing the importance of thorough preoperative planning, particularly regarding specimen extraction in cases involving giant lesions.

In conclusion, we present a rare case of a giant IFP in the terminal ileum that was successfully resected endoscopically. This case highlights the feasibility of using advanced endoscopic techniques, even in anatomically challenging locations such as the terminal ileum. Specifically, the combination of the pocket‐creation method, underwater technique, and lesion anchoring provided stable visualization and adequate maneuverability, enabling safe and complete resection. These strategies may serve as a valuable option for managing similarly large and mobile small intestinal lesions.

## Conflicts of Interest

The authors declare no conflicts of interest.

## Supporting information




**VIDEO S1** Endoscopic submucosal dissection (ESD) for a giant inflammatory polyp in the terminal ileum. Using a traction device attached to the top of the polyp, we pulled the polyp head into the ascending colon and maintained this position by anchoring the device to the opposite side of the ileocecal valve. This stabilization allowed for secure resection of the lesion from the cecal side of its wide stalk using the pocket‐creation method. The dissection was performed under saline immersion. Severe fibrosis was observed directly beneath the lesion, but en bloc resection was successfully completed without intraoperative perforation in 63 min.
